# Reproductive relationships between taxa morphologically close to Elymus caninus (Poaceae: Triticeae)

**DOI:** 10.18699/vjgb-24-02

**Published:** 2024-02

**Authors:** E.V. Shabanova, A.V. Agafonov, O.V. Dorogina

**Affiliations:** Central Siberian Botanical Garden of the Siberian Branch of the Russian Academy of Sciences, Novosibirsk, Russia; Central Siberian Botanical Garden of the Siberian Branch of the Russian Academy of Sciences, Novosibirsk, Russia; Central Siberian Botanical Garden of the Siberian Branch of the Russian Academy of Sciences, Novosibirsk, Russia

**Keywords:** speciation, hybridization, inheritance, taxonomy, Elymus, Poaceae, видообразование, гибридизация, признак, наследование, таксономия, Elymus, Poaceae

## Abstract

A hybridological study of biotypes of species close to Elymus caninus: E. prokudinii, E. viridiglumis, E. goloskokovii, as well as a number of morphologically deviant biotypes in Russia and Kazakhstan, was carried out. The objectives were to study the levels of reproductive relationships and the degree of integration of the species E. goloskokovii, E. prokudinii, and E. viridiglumis into the E. caninus complex. Our estimates of the seed fertility of natural parental biotypes were within 60–90 %. Among the combinations of crossing in F1, the highest seed setting was found in the hybrids formed by parental pairs from close habitats, regardless of the taxonomic rank of biotypes. The highest fertility values (55.6 and 46.1 %) were found in combinations involving E. caninus, E. viridiglumis and E. goloskokovii. It has been concluded that the biotypes of these species included in sexual hybridization form a single recombination gene pool, within which slight differences in reproductive compatibility are observed. The nature of the inheritance of the diagnostic features of lemmas “presence of trichomes” and “length of awns”, according to the digenic and monogenic type, respectively, is shown. The high seed fertility of the created hybrids and the presence of intermediate forms in the F2 generation according to distinctive features indicate the possibility of interspecific introgression when species grow together in natural populations. Thus, the assessment of the inheritance of diagnostic characters makes it possible to classify E. goloskokovii, E. prokudinii, and E. viridiglumis as intraspecific taxa of E. caninus s. l. Data were obtained on the morphological and reproductive properties of interspecific hybrids with the participation of the species E. mutabilis as a possible donor in the speciation of taxa close to E. caninus. In cross combinations of E. caninus × E. mutabilis and E. mutabilis × E. caninus, lower values of seed fertility of hybrids in the F1 and F2 generations were noted compared to hybrids between the species E. caninus, E. goloskokovii, E. prokudinii and E. viridiglumis. Nevertheless, on the basis of chorological and morphological criteria, we concluded that E. caninus and E. mutabilis are independent species.

## Abstract (ru)

Проведено гибридологическое изучение биотипов видов, близких к Elymus caninus: E. prokudinii, E. viridiglumis, E. goloskokovii, а также ряда морфологически отклоняющихся биотипов с территории России и Казахстана. Была поставлена задача изучить уровни репродуктивных взаимоотношений и степени интегрированности видов E. goloskokovii, E. prokudinii и E. viridiglumis в комплекс E. caninus. Значения семенной фертильности природных родительских биотипов оценены нами в пределах 60–90 %. Среди комбинаций скрещивания в F1 наивысшая завязываемость семян выявлена у гибридов, образованных родительскими парами из близких местообитаний, независимо от таксономической принадлежности биотипов. Наивысшие величины фертильности (55.6 и 46.1 %) обнаружены в комбинациях с участием видов E. caninus, E. viridiglumis и E. goloskokovii. Сделан вывод, что биотипы названных видов, включенные в половую гибридизацию, образуют единый рекомбинационный генпул, внутри которого наблюдаются незначительные различия по репродуктивной совместимости. Показан характер наследования диагностических признаков нижних цветковых чешуй «наличие трихом» и «длина остей» по дигенному и моногенному типу соответственно. Высокая семенная фертильность созданных гибридов и наличие в поколении F2 промежуточных форм по различительным признакам свидетельствуют о возможности межвидовой интрогрессии при совместном произрастании видов в природных популяциях. Таким образом, оценка наследования диагностических признаков позволяет классифицировать E. goloskokovii, E. prokudinii и E. viridiglumis как внутривидовые таксоны E. caninus s. l. Получены данные о морфологических и репродуктивных свойствах межвидовых гибридов с участием вида E. mutabilis как возможного донора при видообразовании таксонов, близких к E. caninus. В комбинациях скрещивания E. caninus × E. mutabilis и E. mutabilis × E. caninus отмечены более низкие значения семенной фертильности гибридов в поколениях F1 и F2 по сравнению с гибридами между видами E. caninus, E. goloskokovii, E. prokudinii и E. viridiglumis. Тем не менее на основе хорологического и морфологического критериев нами сделан вывод о видовой самостоятельности E. caninus и E. mutabilis.

## Introduction

Wild cereals of the tribe Triticeae Dumort. (fam. Poaceae Barn.) are of great interest to researchers as possible donors of valuable traits for the main grain crops – wheat, barley and rye. General ideas about the potential possibilities of using wild relatives of wheat to enrich breeding material with new hereditary qualities were first outlined by N.I. Vavilov (1931). Subsequently, the concept of primary, secondary and tertiary gene pools was introduced (Harlan, De Wet, 1971). A similar gene pool system proposed for barley and rye (von Bothmer et al., 1992) includes, in general, some species of perennial grasses.

The genus Elymus L. is the largest genus of the tribe, belonging to the tertiary gene pool, uniting allopolyploid species of perennial grasses with different genomic constitutions. Facultative self-pollination, which promotes the elimination of spontaneous mutations and the consolidation of the consequences of introgressive hybridization, accelerates the processes of morphogenesis and at the same time complicates the systematization of natural populations. In the ongoing microevolutionary differentiation of the genus, the most relevant to study are the phylogenetic relationships between taxa

Elymus caninus (L.) L. is a species with a StStHH genome (Dewey, 1968) and a vast range covering all of Europe from Iceland and the Mediterranean Sea to the Ural Mountains, almost the entire Palearctic part of Siberia, as well as some areas of Central Asia (Tsvelev, 1976; Hultén, Fries, 1986). In Northern Europe, E. caninus is distributed throughout Sweden and Denmark, and somewhat less frequently in Norway and Finland. In Siberia, it is found in almost all areas west of Lake Baikal (Peshkova, 1990). Taking into account the wide distribution of this species and its high adaptability to environmental factors, one can initially assume a noticeable variation in morphological characters within E. caninus. However, E. caninus exhibits low morphological variability compared to other Elymus species. The main reason, in our opinion, is that a number of natural morphotypes with deviating characters have been described as independent species. At the same time, on the one hand, in most cases no evidence was provided for the phylogenetic isolation of new species, and on the other hand, these species were not considered as a component of E. caninus, since they went beyond the limits of its narrow variability. In Russia and Kazakhstan, these species include E. viridiglumis (Nevski) Czer., E. prokudinii (Seredin) Tzvelev (Tsvelev, Probatova, 2019) and E. goloskokovii Kotuch. (Kotukhov, 2004).

The species E. goloskokovii was described from Western Altai (Ivanovsky Range), indicating its wide distribution within the southwestern part of these mountains (Kotukhov, 2004). The protologue notes that E. goloskokovii is a stable fertile hybridogenic species, probably derived from the hybridization of E. fibrosus (Schrenk) Tzvelev and E. trachycaulis (Link) Gould et Shinners, with the possible participation of E. mutabilis (Drob.) Tzvelev. At the same time, the species E. goloskokovii differs from the widespread E. caninus mainly in the character of short (up to 4 mm) awns of lemmas

Elymus viridiglumis was described from the Southern Urals in 1934 based on the collections of S.A. Nevsky as Roegneria viridiglumis Nevski. The species is distributed in the Urals and Western Siberia; it differs morphologically from E. caninus in having hairy or scabrous lemmas. Small populations have also been found in Eastern Kazakhstan.

Elymus prokudinii is an endemic of the subalpine meadows of the forest belt of the Central and Eastern Caucasus, described in 1965 as Roegneria prokudinii Seredin (Seredin, 1965) based on the collections of R.A. Elenevsky. The species is morphologically similar to E. viridiglumis, and differs from it only in its narrow endemic geographic range.

To date, much information has been accumulated indicating that the single recombination gene pool of E. caninus, as a species, is formed not only by typical individuals, but also by a large number of morphologically deviant biotypes (MDBs) that do not correspond to the diagnosis of the species (Gerus, Agafonov, 2006; Agafonov, 2011). In particular, we have obtained evidence that introgressive relationships between E. caninus and E. mutabilis lead to a diversity of transitional interspecific forms (Agafonov, 2013). Since that time, a number of questions remain and new problems have arisen from the perspective of reproductive biology and taxonomy of this vast complex.

One of the most important criteria for the relatedness of living organisms is the ability to produce viable offspring during crossing, which is due to the balanced recombination of genetic material during generations. The study of intra- and interspecific crossbreeding of biotypes makes it possible to model the processes of hybridization and introgression occurring within the genus Elymus. The results obtained from crossing biotypes from close or distant populations make it possible to clarify issues of intraspecific organization, outline the genetic pool and predict the possible course of further speciation pathways. The levels of crossbreeding of biotypes Cs (sexual compatibility) and the fertility of the resulting hybrids are under strict genotypic control and, accordingly, reflect the phylogenetic relationships of the original taxa (Agafonov et al., 2001).

In order to obtain additional data, work was carried out to create and study F1–F2 hybrids between selected biotypes of different taxa of species rank, morphologically close to E. caninus s. l. The objectives were to study the levels of reproductive compatibility of biotypes and the degree of integration of the previously described species E. goloskokovii, E. prokudinii and E. viridiglumis into the E. caninus complex, as well as to supplement data on the morphological and reproductive properties of interspecific hybrids E. caninus × E. mu-tabilis

## Materials and methods

According to G.A. Peshkova (1990), the main diagnostic characteristics of E. caninus include: (1) leaf blades on top with scattered long hairs; (2) glabrate lemmas, rarely with single spines in the upper part; (3) lemmas with straight awns, equal to the lemmas or longer; (4) hairy rachillas.

In addition to the typical morphotype of E. caninus GAT- 9210 and some morphologically deviating biotypes (MDB), biotypes of the above-mentioned species were used in hybridization in eight cross-combinations

Characteristics by which typical individuals of E. caninus and E. mutabilis differ are as follows: long (up to 25 mm) – short (up to 6 mm) lemma’s awns; glabrous – scabrous (hairy) lemmas; the ratio of glume’s length and the adjacent lemma’s length (k = LGl /LLem) is approximately 0.5–0.6 in E. caninus and 0.7–0.8 in E. mutabilis. The last trait must be accompanied by the the presence of membranes at the edges of glumes, which become thinner with increasing k value (the mutabilis type), and conversely, membranes become wider as the k value decreases (the caninus type). This trait is not always clearly identified in most phenotypes due to the presence of a spectrum of intermediate phenotypes in natural populations. The locations of E. mutabilis accessions and species closely related to E. caninus are given in Table 1.

**Table 1.**
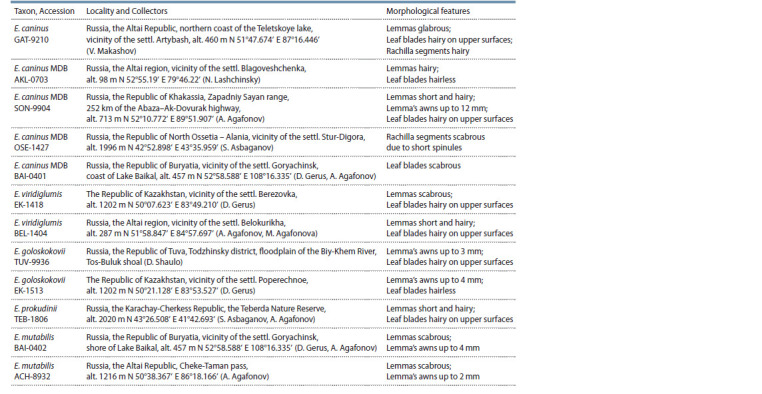
Localization of E. mutabilis accessions and species close to E. caninus, and their morphological features

**Fig. 1.**
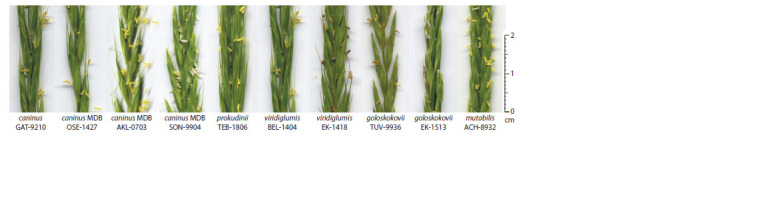
Fragments of flowering spikes of taxa and biotypes close to E. caninus, taken for hybridization

Fragments of flowering spikes of some biotypes of species close to E. caninus are shown in Figure 1. The chasmogamous type of flowering characteristic of all taxa does not prevent the predominant self-pollination of plants, which is supported by the simultaneous maturation of male and female gametophytes, as well as the absence of genetic systems of selfincompatibility (open (bursted) anthers are visible in Fig. 1).

In addition to the diagnostic character “length of the awns of lemmas”, the biotypes included in the hybridization were found to have variability in a number of secondary characters inherent in specific taxa and geographic races: the relative length of the glumes, pubescence of leaf blades (LB), color of anthers, spike density, plant habit and height.

Since to study reproductive compatibility we use seeds of wild plants collected from different points of the species’ ranges, where their morphometric characters largely depend on environmental factors, it is necessary to exclude modification of taxonomic characters. To do this, plants were grown under equalized conditions at the experimental site of the Central Siberian Botanical Garden SB RAS, and only after that their taxonomic affiliation was determined. When selecting parental individuals, forms with different characteristics were selected – plants with glabrous lemmas were crossed with plants that had trichomes on lemmas, plants with short awns of lemmas were crossed with plants with long awns, etc.

The procedures for creating sexual hybrids were carried out using an express method (Lu et al., 1990), which involves preliminary preparation of the ear and manual pollination of each pistil. One of the advantages of this technique is the stimulation of the natural opening of flowers and the simultaneous removal of anthers that have not yet burst. Pollination of each of the several opened emasculated flowers is carried out by the newly burst anther of the father plant, which minimizes the risk of its own or foreign pollen. The use of this technique, with sufficient development, gives more reliable results, since it does not require preliminary emasculation of delicate immature flowers and leads to an increase in the efficiency of hybridization.

Hybridity of F1 plants was confirmed by the presence of characteristics of the paternal plant. The assessment of seed fertility (SF) of plants in generations F1–F2 and levels of sexual compatibility of biotypes (Cs) was carried out according to the principles we developed (Agafonov, 1994; Agafonov, Salomon, 2002). The correspondence of the type of inheritance of morphological characters (presence of trichomes on lemmas and length of lemma’s awns) to Mendel’s laws (monogenic and digenic) in F2 plants was checked using the Pearson criterion (χ2) (Pearson, 1900).

The segregation of the awn length trait in F2 plants was analyzed based on the maximum value of awn length among the spikes of each individual

## Results

Crosses were carried out between species close to E. caninus in six combinations: E. caninus × E. prokudinii (2 combinations), E. caninus × E. viridiglumis, E. goloskokovii × E. caninus, E. caninus × E. goloskokovii, E. goloskokovii × E. viridiglumis; there were also two cross combinations between the biotypes E. caninus and E. mutabilis. From 1 to 3 hybrid grains were obtained in each combination. The results are presented in Table 2.

**Table 2.**
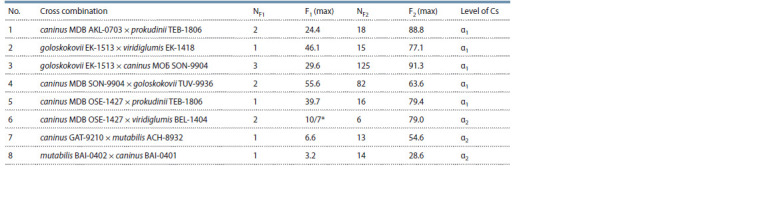
Maximum values of seed fertility in F1 and F2 hybrids (%) and levels of sexual compatibility (Cs) of biotypes among taxa close to E. caninus and in cross combinations of E. caninus × E. mutabilis NF1 and NF2 are the number of analyzed plants in F1 and F2, respectively. * The fraction shows the ratio of the number of completed seeds to the number of spikes from two F1 plants.

The inheritance of morphological characters was assessed in small samples of the F2 generation. Although the sample sizes were not large, they made it possible to assess the degree of discreteness of the trait in phenotypic classes and the nature of their inheritance (Tables 3, 4).

**Table 3.**
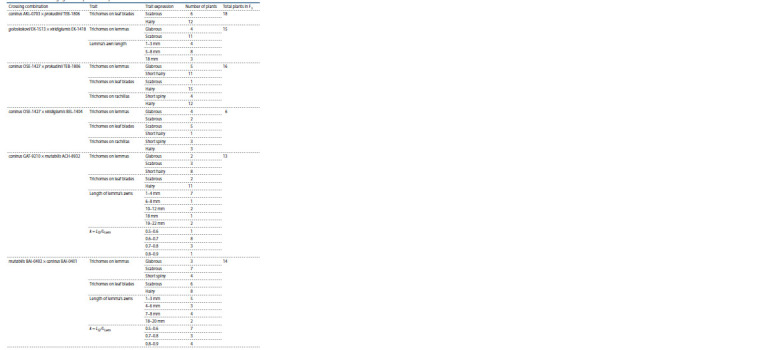
Results of trait segregation in F2 in small samples

**Table 4.**
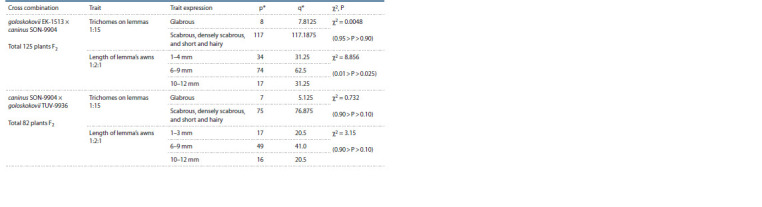
Analysis of trait segregation in the F2 generation in the two largest samples p – actual number of individuals; * q – expected number of individuals.

Pearson’s test χ2 was applied and the level of significance P was assessed for F2 samples in two cross combinations: E. goloskokovii × E. сaninus and E. caninus × E. goloskokovii (see Table 4).

## Discussion

The results of the study showed that the biotypes included in the hybridization, which are close in morphology to the base species E. caninus (see Table 2, No. 1–6), form a single recombination gene pool, within which minor differences in reproductive compatibility are observed. Noteworthy is the relatively small but clear decrease in SF values in F1 hybrids compared to mother plants. This means that all MDBs taken for hybridization belong to the extensive gene pool of E. caninus, but have gone through a certain microevolutionary path in the direction of divergence.

We estimated the SF values of natural biotypes to be within 60–90 %. The maximum values of SF of F1 plants among species close to E. caninus (see Table 2, No. 1–5) were in the range of 24.4–55.6 %, except for the crossing combination of E. caninus × E. viridiglumis (No. 6), where SF was very low; for this reason, the maximum value of SF could not be given. In this combination, out of seven spikes (the total number of spikes in two F1 plants), only 10 seeds were fertile, of which only six were able to grow to the generative stage and produce offspring in F2. In cross combinations between the species E. caninus and E. mutabilis (see Table 2, No. 7, 8), where one F1 plant was obtained, the maximum SF value was in the range of 3.2–6.6 %.

The highest SF values in F1 were found in hybrid plants formed by parental pairs from geographically close habitats (see Table 2): combination 4 from the border territory of the Republic of Khakassia and the Republic of Tuva (E. caninus SON-9904 × E. goloskokovii TUV-9936) – 55.6 %, and combination 2 from North-Eastern Kazakhstan (E. goloskokovii EK-1513 × E. viridiglumis EK-1418) – 46.1 %. Combination 5 from the North Caucasus (E. caninus OSE-1427 × E. prokudinii TEB-1806) was also characterized by a relatively high SF – 39.7 %. This fact also confirms our assumption about the joint microevolutionary path of different taxa within a specific territory (Agafonov, 2011). Moreover, two interspecific combinations (7 and 8), formed by E. caninus and E. mutabilis, showed significantly lower (6.6 and 3.2 %) SF values in the F1 generation than other combinations (1–5). As for the hybridization variant E. caninus OSE-1427 × E. viridiglumis BEL-1404 (6), low SF values in the F1 hybrid can be explained by the significant geographic isolation of the original parental biotypes.

Generally, the values of seed fertility in F2 represent a range of variability within certain limits, determined, among other things, by the degree of phylogenetic proximity of the parental biotypes. In all crossing combinations, an increase in SF values in F2 was observed (see Table 2), which is associated with the normalization of genetic recombination according to the RGP principle. The levels of sexual compatibility of biotypes in almost all crossing variants (except for two involving E. mutabilis and variant 6) correspond to free genetic recombination (α1). The possibility of interbreeding and restoration of seed productivity over several generations indicates the presence of a homologous part of the genome in the parental forms. Such hybridization can be considered as natural seed propagation of two different forms of the same species.

Based on the presented results of hybridization, it was concluded that the nature of inheritance of traits during the segregation of hybrids in F2 in self-pollinating taxa depends on the parental genotypes, which can differ in a different number of loci and alleles (one or more), forming the general gene pool of the taxon. In these crossing combinations, the nature of inheritance of the trait “presence of trichomes on lemmas” was determined to be digenic, while the inheritance of the trait “awn length” was determined to be monogenic with incomplete dominance.

The distribution of phenotypes according to the trait “presence of trichomes on lemmas” by class allowed us to assume a segregation of 1:15, which corresponds to digenic inheritance. The level of significance test (P) was low in some cases, which may be explained by the insufficient number of F2 plants forming the sample. However, based on the segregation of phenotypes, one can roughly assume the type of inheritance of diagnostic traits.

Previously, we conducted expeditionary collections of species close to E. caninus in the Republics of Altai and Tuva, the Caucasus, and Eastern Kazakhstan. The samples were studied experimentally. Let us give a brief overview of the most significant results for this article. Since small populations of E. viridiglumis were found to occur in Eastern Kazakhstan, we hypothesized possible routes for the formation of the species in this territory (Agafonov, 2013). One cannot but agree with the comment that according to spike characteristics, some biotypes of E. viridiglumis are similar to E. komarovii (Tsvelev, Probatova, 2019); however, the genetic distance of these two species was experimentally shown (Agafonov et al., 2017). A taxon similar to E. goloskokovii in the character “short awns of lemmas” was described from Northern Europe as E. caninus var. muticus (Holmb.) Karlsson. We studied the living material of this taxon in an experiment (Gerus, Agafonov, 2006), and it was concluded that this species has a polyphyletic hybrid origin.

ISSR analysis using a wide range of samples of species morphologically similar to E. caninus from different localities within Russia showed that the species E. viridiglumis, E. prokudinii and E. goloskokovii represent groups of individuals that are also phylogenetically close to E. caninus (Shabanova (Kobozeva) et al., 2020). The assumption was confirmed that E. viridiglumis has a polyphyletic origin, as a result of microevolutionary processes in populations of E. caninus s. l., possibly with the participation of E. mutabilis. For the Caucasian endemic E. prokudinii and the Kazakh endemic E. goloskokovii, origin is also assumed to be a result of introgression or spontaneous mutagenesis, i. e. manifestations of natural intraspecific polymorphism of E. caninus. The remoteness of E. fibrosus from all taxa phylogenetically close to E. caninus cast doubt on the assumption of the origin of E. goloskokovii from the hybridization of E. fibrosus and E. trachycaulis, especially considering the introduced North American origin of the latter (Agafonov, Baum, 2000). Taking into account our early studies of species close to E. caninus (Agafonov, 2011), it was concluded that the taxa E. viridiglumis, E. prokudinii and E. goloskokovii are not phylogenetically separate and should be transferred to the intraspecific rank of E. caninus s. l.

Reproductive relationships between Elymus caninus and E. mutabilis

The typical morphotypes of E. caninus and E. mutabilis have a special character of reproductive relationships. Previously, we touched upon the topic of the influence of E. mutabilis on speciation in Eastern Kazakhstan (Gerus, Agafonov, 2006), including in comparison with data on intraspecific variability of E. caninus (Agafonov, 2011). Some E. caninus × E. caninus hybrids were found to have lower compatibility than E. caninus × E. mutabilis hybrids.

In this study, the parental biotypes of E. caninus and E. mutabilis had typical characteristics of these species; differences were identified only between biotypes of E. caninus in the pubescence of the upper surfaces of leaf blades: hairy surfaces were noted in the parental biotype GAT-9210, scabrous (hairless) ones were noted in the BAI-0401 biotype. Data on SF and the segregation of morphological traits’ characters in F2 hybrid samples in two cross combinations are given in Tables 5 and 6.

**Table 5.**
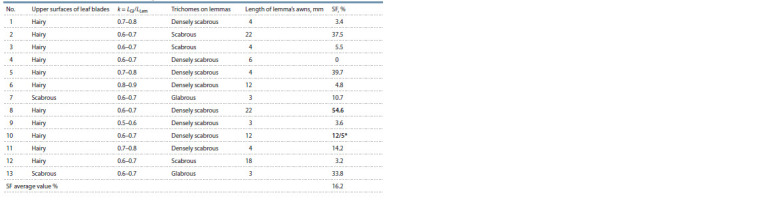
Main morphological characteristics and seed fertility (SF) in the F2 sample of the E. caninus GAT-9210 × E. mutabilis ACH-8932 hybrid

**Table 6.**
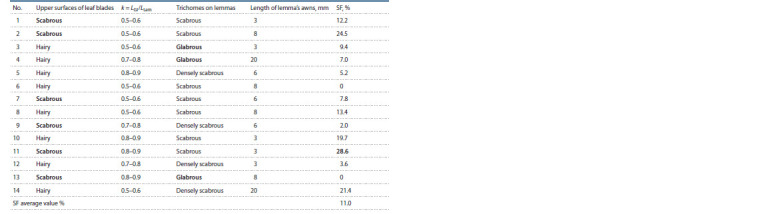
Main morphological characteristics and seed fertility (SF) in the F2 sample of the E. mutabilis BAI-0402 × E. caninus BAI-0401 hybrid The fraction shows the ratio of the number of completed seeds to the number of spikes from two F1 plants.

Elymus caninus GAT-9210 × E. mutabilis ACH-8932. In the grown F2 sample of 13 individuals (see Table 5), only one absolutely sterile plant (4) was noted; the highest SF value was 54.6 % (plant 8). This value was slightly lower than in all combinations involving taxa close to E. caninus (see Table 2). In addition, a wide range of SF values was noted, which is a characteristic feature for relatively distant hybrids. Based on a set of morphological characters, two individuals (2 and 8) were recombinant ones (see Table 5), and corresponded to the diagnosis of E. viridiglumis (hairy upper surfaces of leaf blades, scabrous lemmas and long awns up to 18–22 mm). This fact confirms our assumption about the polyphyletic origin of the abovementioned taxon. Moreover, only two individuals had scabrous upper surfaces of leaf blades (plants 7 and 13), the rest had hairy surfaces to a greater or lesser extent (11 individuals). This fact may indicate a small number of alleles by which the parental individuals differed, since during segregation in F2 for one discriminating allele, there is a greater probability of obtaining 2 individuals with recessive homozygotes per sample of 13 plants (1 out of 4) than with two alleles (1 out of 16).

Based on the diagnostic trait of lemma’s awn length, we identified morphotypes with six values from 3 mm (morphotype E. mutabilis) to 22 mm (morphotype E. caninus). The large number of phenotypes for this trait can probably be explained by the fact that each allele makes an additive contribution to the formation of the awn length trait. And the greater the difference in the length of the awns in the parental forms, the greater the range of variability in the offspring. In addition, the value of this quantitative characteristic is quite difficult to fix

Elymus mutabilis BAI-0402 × E. caninus BAI-0401. The F2 sample in this combination is represented by 14 individuals with an average SF value of 11.0 % (see Table 6), which is the smallest value among all studied samples. At the same time, the maximum value of SF in F2, 28.6 %, was also the smallest compared to other samples (see Table 2). In the sample, six individuals with completely naked leaf blades were noted (see Tables 3, 6). This may mean that the parental biotype of E. mutabilis BAI-0402 was heterozygous for the allele(s) controlling the trait. At the same time, three individuals with glabrous lemmas (morphotype E. caninus) differed from each other in the length of lemma’s awns (see Table 6). That is, potentially new taxa have emerged if traditional classification methods are followed. Despite the fact that individual 13 was completely sterile, the other two (3 and 4) had, although reduced, quite sufficient seed fertility (9.4 and 7.0 %) for their own reproduction and consolidation in the next generations.

**Fig. 2.**
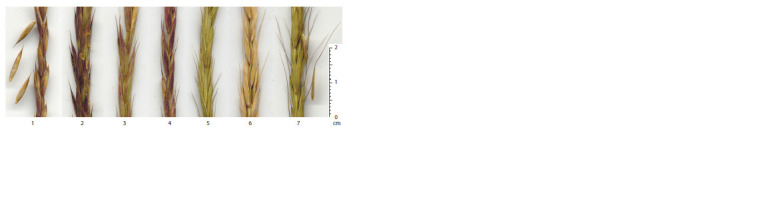
Main morphological characteristics and seed fertility (SF) in the F2 sample of the E. caninus GAT-9210 × E. mutabilis ACH-8932 hybrid The fraction shows the ratio of the number of completed seeds to the number of spikes from two F1 plants.

In a small part of the overlapping areas of E. caninus and E. mutabilis within the mountainous regions of the Republic

of Khakassia (Krasnoyarsk Territory), we found biotypes with extreme and all intermediate values of the trait “lemma’s awn length” (Fig. 2). This phenomenon can only be explained by acts of mutual introgression between the two species, as well as multiple allelism of the genes that control this trait. The existence of the introgression mechanism is confirmed by the SF values in interspecific hybrids (see Tables 5, 6).

The hybrids we created in E. caninus × E. mutabilis combinations had reduced seed fertility at the level of α2. This level of SF certainly reduces the competitive ability in natural conditions, but the probability of the formation of descendants and their consolidation in populations is quite high. In general, the increase in SF in E. caninus × E. mutabilis hybrids to a normal level already in the F2 generation confirms the possibility of a fairly easy exchange of genetic material between species. This means that some spontaneous hybrids in natural conditions have a chance to survive in subsequent generations, while increasing the overall population biodiversity, as has been shown previously (Sun et al., 1999).

## Conclusion

Thus, based on the indicators of interbreeding and character segregation among taxa close to the widespread species E. caninus, an integral assessment of the relationships between biotypes was obtained. From the results of the study, the feasibility of a taxonomic revision logically follows. In our opinion, the taxa currently recognized as independent species E. prokudinii, E. viridiglumis and E. goloskokovii due to their polyphyletic origin must be transferred to the intraspecific rank within the polymorphic species Elymus caninus s. l. The main confirmation of the phylogenetic unity of these taxa is the high values of SF already in the first generation of hybrids and free recombination of diagnostic characters (reproductive compatibility) at the α1 level.

Based on the results of chorological analysis and hybridization of selected biotypes of Elymus mutabilis and E. caninus, it was concluded that E. mutabilis is an independent species with the widest range in the Northern Hemisphere and with high intraspecific variability in many characters.

## Conflict of interest

The authors declare no conflict of interest.
